# J‐SUPPORT research policy for oral mucositis associated with cancer treatment

**DOI:** 10.1002/cam4.4811

**Published:** 2022-06-12

**Authors:** Tomoya Yokota, Takao Ueno, Yoshihiko Soga, Hiroto Ishiki, Yasuhito Uezono, Takehiko Mori, Sadamoto Zenda, Yosuke Uchitomi

**Affiliations:** ^1^ Division of Gastrointestinal Oncology Shizuoka Cancer Center Sunto‐gun Japan; ^2^ Department of General Internal Medicine, Dentistry, Oncologic Emergency National Cancer Center Hospital Tokyo Japan; ^3^ Division of Hospital Dentistry Okayama University Hospital Okayama Japan; ^4^ Department of Palliative Medicine National Cancer Center Hospital Tokyo Japan; ^5^ Department of Pain Control Research The Jikei University School of Medicine Tokyo Japan; ^6^ Supportive and Palliative Care Research Support Office National Cancer Center Hospital East Kashiwa Japan; ^7^ Project for Supportive Care Research, Exploratory Oncology Research and Clinical Trial Center National Cancer Center Kashiwa Japan; ^8^ Department of Hematology Tokyo Medical and Dental University Tokyo Japan; ^9^ Department of Radiation Oncology National Cancer Center Hospital East Kashiwa Japan; ^10^ Innovation Center for Supportive, Palliative and Psychosocial Care National Cancer Center Hospital Tokyo Japan

**Keywords:** cancer treatment, clinical trial research policy, oral mucositis, supportive care

## Abstract

**Background:**

Oral mucositis is one of the main areas of research in supportive and palliative care of cancer patients. However, the methodology of prospective clinical trials on oral mucositis has not been established, despite its uniqueness. Here, we propose a novel research policy on oral mucositis, including an implementable set of recommendations for researchers conducting clinical trials.

**Methods:**

The first draft was developed by an expert panel of six specialists from the Japanese Supportive, Palliative, and Psychosocial Care Study Group. A provisional draft was developed after review by the following medical societies: the Japanese Association of Supportive Care in Cancer, the Japanese Association of Oral Supportive Care in Cancer, the Japanese Cancer Association, and the receipt of public comments.

**Results:**

The research policy on oral mucositis mainly consists of the following components: (i) definition of oral mucositis; (ii) characteristics of oral mucositis; (iii) characteristics of oral mucositis research; (iv) target population for oral mucositis research; (v) endpoints and assessment measures in oral mucositis; (vi) eligibility criteria; (vii) research design; (viii) minimally recommended intervention in oral mucositis research as a supplement. The final policy (Ver1.0) was completed on August 16, 2021.

**Conclusions:**

This policy may serve as a significant reference for planning and conducting clinical trials for the management of oral mucositis.

## Abstract 2

An expert panel of the Japanese Supportive, Palliative, and Psychosocial Care Study Group (J‐SUPPORT) developed a specific and precise policy for clinical studies on oral mucositis induced by cancer therapy. The contents of this policy were systematically organized according to the items in the study protocol, including endpoints, eligibility criteria, assessment measures, and research design. This policy is useful for the researchers in the field of oral mucositis research to conduct high‐quality clinical studies and report reliable evidence.

## 1 INTRODUCTION

Oral mucositis (OM) is extremely common toxicity occurring after chemotherapy and radiotherapy for cancer.^1^, ^2^ There are currently huge unmet needs for management interventions for OM. Therefore, OM is one of the main areas of focus in supportive and palliative care research on cancer treatment. Several prospective studies have been conducted to investigate the efficacy of pharmacotherapies and medical interventions, as well as noninvasive or minimally invasive interventions. However, the methodology of prospective clinical trials on OM is unique compared to those on cancer treatment, in terms of multidisciplinary approaches, study design, setting of the target population, and endpoints.

Recently, a research policy was established by the Japanese Supportive, Palliative, and Psychosocial Care Study Group (J‐SUPPORT) in Japan, providing a basic common framework for clinical research regarding supportive and palliative care.^3^ Based on this universal policy, a panel of experts in the field of OM research developed a specific and precise policy for clinical OM studies. In this policy, we attempted to collate literature and clinical experience to establish an implementable set of recommendations for researchers to design and conduct clinical research on OM.

## 2 MATERIALS AND METHODS

### 2.1 Aim

The aim of this policy is to demonstrate the basic policy for clinical research on OM in supportive and palliative care for cancer. Specifically, this policy provides (1) a definition of OM, (2) characteristics of OM, (3) characteristics of OM research, (4) risk factors for OM, (5) target population for OM research, (6) endpoints and assessment measures in OM research, (7) eligibility criteria, and (8) research design. Minimally recommended interventions for OM research are also described.

### 2.2 An expert panel of OM‐related research policy

The development of the research policy for OM was initiated by an expert panel within the Japanese Supportive, Palliative, and Psychosocial Care Study Group (J‐SUPPORT; https://www.j‐support.org/) in November 2019. The expert panel included six experts: one medical oncologist, two dentists, one palliative care physician, one hematologist, and one basic researcher.

### 2.3 First draft

The backbone of the policy was developed through daily discussions via e‐mail and web conferences. First, we discussed the components and detailed items of our research policy. The members of the expert panel subsequently identified relevant items based on their expertise. The first draft was completed in November 2020 (Version 0.1).

### 2.4 Provisional draft

The J‐SUPPORT policy working group modified the first draft to establish a provisional draft from November to December 2020. The first draft was critically reviewed by the following medical societies: the Japanese Association of Supportive Care in Cancer, the Japanese Association of Oral Supportive Care in Cancer, and the Japanese Cancer Association. Public comments were invited on the provisional draft. The J‐SUPPORT policy working group modified the first draft according to the public's comments to establish the first edition (Version 1.0) on August 16, 2021.

## 3 RESULTS

This section describes the research policy in the area of OM associated with cancer treatment.

### 3.1 Definition of OM in this research policy

In this policy, OM is specifically defined as an injury to the oral mucosa associated with cancer treatment, including cancer pharmacotherapy and radiotherapy (RT) (primary OM). Hematopoietic stem cell transplantation also leads to an increasing incidence and prevalence of OM as mucosal toxicity of conditioning. In the setting of allogeneic hematopoietic stem cell transplantation, however, chronic graft‐versus‐host disease (GVHD) could involve oral mucosa with different pathophysiology. Furthermore, criteria for diagnosing and scoring the severity of chronic GVHD have been established by the National Institutes of Health Consensus Conference, but they refer not only to oral symptoms but also other organ disorders.^4^, ^5^ Therefore, this policy does not target the research area of chronic GVHD‐related OM. Furthermore, OM in terminally ill cancer patients is excluded from the research area in this policy because OM could occur in the absence of cancer pharmacotherapy and RT.

### 3.2 Type of OM

#### 3.2.1 OM associated with RT

Irradiation of the head and neck region is substantially associated with an increase in OM because the oral cavity is included in the irradiated field. Specifically, OM is unavoidable in patients with head and neck cancer (HNC) or malignant lymphoma who are scheduled to receive >50 Gy irradiation in the pharyngeal or oral space. OM develops in more than 90% of patients receiving chemoradiotherapy (CRT) for HNC. The severity of radiation‐induced OM depends on radiation dose and field. Therefore, the radiation dose and field must be confirmed in planning clinical studies.^6^

In the treatment of HNC, the OM severity induced by RT is considerably enhanced by the concurrent use of cytotoxic^7^, ^8^ and targeting agents (cetuximab).^9^

Damage to the salivary glands by irradiation causes a dry mouth and facilitates bacterial proliferation inside the oral cavity through a decrease in mucosal resistance and self‐purification.

#### 3.2.2 OM associated with pharmacotherapy (cytotoxic agents and molecular targeting agents)

Cytotoxic chemotherapeutic agents that induce myelosuppression can promote OM.^10^ High‐dose chemotherapy with or without total‐body irradiation frequently causes OM in stem cell transplantation.^11^, ^12^, ^13^ Molecular targeting agents are classified into the following groups according to this policy: (1) inhibitors of mammalian target of rapamycin (mTOR),^14^ (2) tyrosine kinase inhibitors (TKIs), (3) immune checkpoint inhibitors, and (4) other antibodies or molecular targeting agents. OM can occur as a severe drug‐related symptom of Stevens‐Johnson syndrome.

#### 3.2.3 OM associated with chronic GVHD

OM associated with chronic GVHD is excluded from the research area in this policy.

#### 3.2.4 OM at the end‐of‐life care

OM in end‐of‐life cancer is excluded from the research area in this policy.

### 3.3 Characteristics of OM

#### 3.3.1 Common mucosal findings

OM is commonly characterized by erythema, erosion, and ulceration of the oral mucosa.

#### 3.3.2 Local impact of OM

Cancer pharmacotherapy and RT affect the homeostasis of normal epithelial cells in the oral cavity.^1^ Loss and damage of these epithelial cells result in mucosal atrophy, necrosis, and ulceration. This destruction of the oral mucosal barrier allows bacteria to enter the host directly, leading to opportunistic local infections in the mouth.

#### 3.3.3 Systemic impact of OM

OM is associated with gastrointestinal symptoms such as oral pain, taste changes, and nausea. OM subsequently causes poor oral hygiene and difficulties in eating and swallowing. These factors collectively increase the susceptibility of patients to oral and systemic infections, including aspiration pneumonitis, febrile neutropenia, and bacteremia, especially in immunosuppressed patients. Oral pain, difficulty in oral intake, and dysarthria induced by OM impair patients' quality of life (QOL).

#### 3.3.4 The impact of OM on cancer treatment

The occurrence of OM has a significant impact on the completion of cancer treatment.^15^, ^16^ OM is dose‐limiting toxicity associated with treatment delay and interruptions and dose reductions of RT, pharmacotherapy, and a combination of RT and pharmacotherapy. The detrimental effect of OM on treatment compliance may ultimately affect therapeutic outcomes, including cure rates, remission durability, and patient survival.^1^, ^17^

### 3.4 Characteristics of OM research

There is a strong demand for the prevention and treatment of OM in supportive care for cancer. However, because there are a number of confounding risk factors involved in the OM onset, as described in Section 3.5, and inter‐patient variability in patient characteristics, it is challenging to standardize the target population in clinical trials of OM.

Because the basic structure of the oral mucosa is simple, there is a similarity in the phenotype of OM regardless of its cause, even though OM is induced by various kinds of risk factors, including host‐ and cancer treatment‐related factors. Therefore, it is difficult to determine endpoints and assessment measures in OM research. Multiple factors are associated with eating and breathing disorders caused by OM. For example, salivary gland disorders and subsequent dry mouth, dysgeusia, and dysphagia collectively influence eating and breathing functions. Therefore, evaluating these functional aspects is often challenging.

In the management of OM, a multidisciplinary approach involving physicians, nurses, pharmacists, dentists, dental hygienists, dieticians, speech therapists, and physical therapists is required. Therefore, the inter‐institutional variability in the quality of interventions is notable.

Supportive care aims to prevent and reduce the occurrence of OM during cancer treatment. However, even though a novel intervention can prevent OM and relieve OM‐related symptoms caused by cancer treatments, the detrimental effect of such interventions on cancer treatment should be avoided. Therefore, it is critical to consider the balance between cancer treatment and supportive care for adverse oral reactions. For instance, regular use of nonsteroidal anti‐inflammatory drugs (NSAIDs) for pain control may increase the risk of cisplatin (CDDP)‐induced nephrotoxicity in HNC patients undergoing CRT with concurrent CDDP, which may subsequently impede CDDP administration.

Lastly, the duration of a study on OM should be determined in accordance with the type of cancer treatment and the anticipated duration of OM.

### 3.5 Risk factors for OM

Information on the risk factors for OM should be collected from medical records. The risk factors associated with susceptibility to OM include both host‐ and cancer treatment‐related factors, as follows.

#### 3.5.1 Host‐related factors

1. Poor oral hygiene: insufficient plaque control and oral clearance
2. Possible mucosal injury factors: sharp edge in the tooth (e.g., caries), malalignment of the tooth, and ill‐fitting denture
3. Dry mouth: caused by salivary gland disorders (including a decrease in salivary serosity)
4. Immune suppressive status: peripheral blood cell count, aging, regular use of steroids, and comorbidity of diabetes mellitus
5. Poor nutritional condition in association with the delay in the mucosal healing process
6. Smoking history: Direct oxidative stress and/or activation of signal transduction pathways may be associated with underlying mechanisms. Nicotine plays a role in decreasing blood flow and vasoconstriction in the oral mucosa.^18^, ^19^, ^20^, ^21^

#### 3.5.2 Cancer treatment‐related factors

1. Irradiated field, dose, fraction, and concurrent pharmacotherapy are risk factors for OM in RT of the head and neck region.
2. Pharmacotherapy, including cytotoxic chemotherapeutic agents (fluoropyrimidines, methotrexate, taxan, anthracycline, etc.) and molecular‐targeting agents
3. The anticholinergic drug uses

### 3.6 Target population for OM research

It is highly recommended that the content of cancer treatments should be as homogeneous as possible within the study cohort. If different treatment regimens are used in randomized trials, the factors regarding cancer treatments should be set as stratification factors and should be well balanced between the control and experimental arms.

#### 3.6.1 Patients undergoing RT in the head and neck region

In the treatment of RT, the presence or absence of concurrent pharmacotherapy greatly influences OM severity. For instance, the extent of acute toxicity produced by CRT is considerably higher than that of RT alone owing to the intensified local host tissue response.^22^ Therefore, patients who receive CRT and RT alone should not be the same population target. Similarly, the radiation technique should be the same in a target population. For instance, patients receiving electromagnetic waves and those receiving particle beams should not be the same population target. Furthermore, concurrent pharmacotherapy in combination with RT should be the same within the study cohort. For instance, patients receiving CRT with concurrent CDDP and those receiving a combination of cetuximab, an epidermal growth factor receptor‐targeting monoclonal antibody, and radiation therapy should not be the same population target.

#### 3.6.2 Patients treated by pharmacotherapy

As the OM frequency may differ among the types of pharmacotherapy, the dose and dosing schedule of pharmacotherapy should be the same within a target population.

#### 3.6.3 Recipients of hematopoietic stem cell transplantation

In hematopoietic stem cell transplantation, the frequency and severity of OM are largely influenced by the content and dose of chemotherapy used for conditioning, presence or absence of total body irradiation, and dose. Therefore, the content of chemotherapy and total body irradiation should be the same in the target population in hematopoietic stem cell transplantation recipients.

### 3.7 Endpoints and assessment measures in OM research

#### 3.7.1 General remarks

There are two types of assessment measures to determine the OM severity: clinician‐reported outcomes (CRO) and patient‐reported outcomes (PRO). CRO are objective measurements evaluated by medical staff, whereas PRO are subjective measurements used to assess adverse symptomatic events that occur directly from patients' responses, such as oral intake and pain. OM‐related symptoms considerably worsen the QOL through swallowing dysfunction, poor oral intake, and dysarthria. Therefore, an assessment of QOL, as well as that of the objective severity of OM, is highly recommended.

In randomized trials, it is mandatory to use common assessment measures between the study arms. Specifically, observers of OM and the procedure of OM measurement should be unified between the arms. Furthermore, the observation period and frequency should be stipulated in the protocol.

#### 3.7.2 Assessment measures in the clinical study of OM

Assessment measures for OM severity in the clinical study were classified into the following categories: (1) assessment of OM severity, (2) compliance with cancer treatment and treatment outcomes, (3) contents of secondary supportive care and medical resources for OM, (4) overall adverse events other than OM, and (5) QOL assessment. Primary endpoints should be set, depending on the timing and aspects that investigators aim to improve the outcome of OM in the study.

##### Assessment of OM severity

The OM severity was assessed by (1) medical staff (clinicians) and (2) patients. The following are the features of each assessment scale.

###### Clinician‐Reported Outcomes: CROs

####### Introduction of CRO assessment measures

CROs are objective assessment measures that are classified into two categories: functional disorders and symptoms induced by OM, and mucosal findings. The former reflects oral intake and swallowing function, while the latter is associated with epithelial defects in the oral cavity and the subsequent risk of infection. Therefore, both assessment measures are important in OM research of cancer patients. Common Terminology Criteria of Adverse Events (CTCAE), World Health Organization (WHO), Oral Mucositis Assessment Scale (OMAS), and Radiation Therapy Oncology Group (RTOG) scale are commonly used to grade severity (Table 1). However, the degree of importance placed on the functional/symptomatic aspects of OM or mucosal findings differ among the scales (Table 2). Therefore, which endpoints are chosen by investigators depends on the primary purpose of the clinical trials.

**TABLE 1:** Assessment scales for the severity of oral mucositis in the clinical study

Assessments	Adverse event	Grade			
		1	2	3	4
CTCAE v3.0	Mucositis/stomatitis (clinical exam)	Erythema of the mucosa	Patchy ulcerations or pseudomembranes	Confluent ulcerations or pseudomembranes; bleeding with minor trauma	Tissue necrosis; significant spontaneous bleeding; life‐threatening consequences
	Mucositis/stomatitis (functional/symptomatic)	Minimal symptoms, normal diet; minimal respiratory symptoms but not interfering with the function	Symptomatic but can eat and swallow‐modified diet; respiratory symptoms interfering with function but not interfering with ADL	Symptomatic and unable to adequately aliment or hydrate orally; respiratory symptoms interfering with ADL	Symptoms associated with life‐threatening consequences
CTCAE v4.0	Mucositis oral	Asymptomatic or mild symptoms; intervention not indicated	Moderate pain; not interfering with oral intake; modified diet indicated	Severe pain; interfering with oral intake	Life‐threatening consequences; urgent intervention indicated
CTCAE v5.0	Mucositis oral	Asymptomatic or mild symptoms; intervention not indicated	Moderate pain or ulcer that does not interfere with oral intake; modified diet indicated	Severe pain; interfering with oral intake	Life‐threatening consequences; urgent intervention indicated
WHO^a^		Soreness, with or without erythema	Erythema, ulcers; still able to swallow solid foods	Ulcers with extensive erythema; unable to swallow solid foods	Ulcers with extensive erythema; alimentation not possible
OMAS^b^	Ulceration/Pseudomembrane (cm^2^)	< 1	1–3	>3	–
	Erythema	Moderate	Pronounced	–	–
RTOG Scoring Criteria		Irritation, may experience slight pain, not requiring analgesic	Patchy mucositis that may produce inflammatory serosanguinitis discharge; may experience moderate pain requiring analgesia	Confluent, fibrinous mucositis, may include severe pain requiring narcotic	Ulceration, hemorrhage, or necrosis

^a^ World Health Organization Oral Toxicity Scale Grading of Oral Mucositis.

^b^ Oral Mucositis Assessment Scale.

**TABLE 2:** Comparison of assessment scales for the severity of oral mucositis

	CTCAE v3.0	CTCAE v4.0	CTCAE v5.0	WHO	OMAS
Description on function/ symptom	Oral intake	Pain and oral intake	Pain and oral intake	Pain and oral intake	Not described
Description on mucosal findings	Erythema, ulceration, pseudomembranes, hemorrhage	Not described	Ulceration	Erythema and ulceration	Precise scoring system according to anatomical sites

Assessment of OM severity by OMAS primarily focuses on mucosal findings to provide a grade for objective measurement. Clinicians need to rate the extent of ulceration (0–3) and erythema (0–2) in nine intraoral locations, namely, the upper and lower lips, right and left cheek, right and left ventral and lateral tongue, the floor of the mouth, soft palate/fauces, and hard palate.^23^, ^24^

Assessment by the CTCAE ver. 3.0, WHO, and RTOG is a combination of mucosal findings and functional/symptomatic aspects. Therefore, the use of OMAS, CTCAE version 3.0, RTOG, or WHO is recommended if investigators aim to assess the oral mucosal outcome as a consequence of intervention for the local oral cavity based on the clinical examination of mucosal findings. By contrast, the main focus of the assessment by CTCAE version 4.0 and version 5.0 is a symptom and functional disorder induced by OM, including pain or oral intake disability. CTCAE version 4.0 and version 5.0 are frequently available in determining the overall outcome in supportive care against OM, whereas these measurement tools may be insufficient for evaluating mucosal findings. It should be noted that pharyngeal pain induced by local oral intervention may be misestimated as that induced by OM in the assessment by the symptomatic scale.

RTOG has developed acute radiation morbidity scoring criteria for the evaluation of RT treatment.^25^ The purpose of RTOG grading is to specifically evaluate acute toxicities induced by irradiation, but not by pharmacotherapy or GVHD. RTOG grading relies on a clinician's ability to judge the anatomical changes associated with OM (size and characteristics of ulceration).

The Revised Oral Assessment Guide (rOAG) and Oral Health Assessment Tool (OHAT) are comprehensive assessment tools for the inside of the oral cavity but are not direct assessment measures for OM. Therefore, it is recommended that the use of rOAG and OHAT as endpoints be limited.

####### Method of CRO assessment

The OM severity is described from the following aspects: (1) frequency of all grades of OM and (2) frequency of OM of more than a pre‐specified grade. Assessments along the axis of time included (3) the worst grade of OM during the observation period, (4) the time to occurrence of OM, and (5) the duration of OM. The study protocol should clarify these endpoints, the data analysis plan, and their interpretation.

Objective assessment of mucosal findings is challenging due to the difficulty in objective quantification, but it is critical to improve the quality of clinical studies. Notably, there is inter‐and intra‐observer variability in the diagnosis of OM for the following reasons.

First, clinical examination of the oral cavity is technically difficult, particularly in patients with oral pain and trismus. Medical staff involved in OM evaluation should undergo specific training and be qualified, if possible, to minimize inter‐observer variation and familiarize themselves with the OM measurement scales.^26^ The number of OM observers was limited by careful selection.

Second, the grading determined by the investigators involved in the observation of the oral mucosa may be highly biased. Depending on the patient's complaint of oral pain or disability of oral intake, investigators' bias may overestimate mucosal findings. To ensure assessment accuracy and consistency, the organization of an independent central review committee (ICRC) is highly recommended. ICRC members should be independent of the observers of the oral mucosa. In the process of central review, photographic data of the oral mucosa uploaded by investigators or the Oral Mucositis Assessment Sheet may be available. The assessment sheet is organized for clinical examination, on which the condition of the oral mucosa at separate sites in the oral cavity is systematically scored.^27^ Importantly, ICRC members should be blinded to patient clinical outcome data, including the presence or absence of experimental intervention, and perform OM grading simply based on such objective oral information. Furthermore, observation of the oral mucosa and subsequent determination of OM grading should be performed separately. For instance, observers of the oral mucosa mechanically score each mucosa‐related variable on the Oral Mucositis Assessment Sheet but are not involved in overall OM grading. Thereafter, the assessment sheets should be sent to the ICRC to automatically and separately determine the OM severity, based on the check sheet.

Finally, when photographic data of the oral mucosa are utilized as visual information, the results of the assessment may vary under different shooting conditions in the photographic technique. To maintain consistent photographic quality, the procedure of taking photographs should be standardized in the protocol.

Taken together, systematic oral observation procedures should be clarified in the protocol or standard operating procedures to standardize the evaluation of CROs.

###### Patient‐Reported outcomes: PROs

####### Introduction of PRO assessment measures

PRO is defined as any symptom or status of a patient's health condition that comes directly from the patient, without interpretation of the patient's response by healthcare providers. The PRO measurement items in clinical trials of OM include pain, swallowing difficulty, dry mouth, and taste changes.

The NCI developed a library of PRO items to supplement CTCAE, called PRO‐CTCAE. In total, PRO‐CTCAE contains 124 individual items representing the 78 symptomatic AEs^28^, ^29^ (http://www.jcog.jp/doctor/tool/PRO_CTCAE.html). Our expert panel recommended collecting patient self‐reports using PRO‐CTCAE. All items were individually selected from the PRO‐CTCAE according to the purpose of each clinical trial.

Pain measurements should be subdivided into those at rest, in conversation, and oral intake or swallowing. The visual analog scale (VAS), numeric rating scale (NRS), face scale, and support team assessment schedule (STAS) are used to assess pain induced by OM. However, evidence regarding the correlation between OM severity and NRS/VAS scores has not been established.

####### Method of PRO assessment

The Consolidated Standards of Reporting Trials (CONSORT) PRO provides guidance on the reporting of PROs in randomized trials.^30^ Standard Protocol Items: Recommendations for Interventional Trials (SPIRIT)‐PRO is an international, consensus‐based, PRO‐specific protocol guidance in which recommendations for items that should be addressed and included in clinical trial protocols are provided.^31^ There is a possibility that the difference in PRO between arms in randomized trials is changeable, depending on the specific time point of measurements and its frequency. For instance, PRO at the time of measurement may be quite different from that during the past week or at the time point when the symptom is worst. Furthermore, several methods of analysis could be applied within one randomized trial, such as a comparison of the worst grade in the observation period, a time to deterioration of PRO compared to baseline, analysis of between‐group differences in overall means, or an interval before reaching a particular threshold of symptoms.^32^ The inconsistencies in PRO measurements between arms could produce contradictory results and affect the overall risk or benefit assessment of oral supportive care. In our recent meeting, there was a clear consensus that the method of PRO measurements should be the same between the study arms, and the data analysis plan should be clearly specified in the protocol when designing the trial.

PRO measurements of oral intake and swallowing function may be modified by multiple factors. For instance, dry mouth, dysgeusia, and dysphagia often significantly influence the functions and symptoms associated with OM.

PRO assessment measures should be used in accordance with the manual. Capturing and editing of validated PRO assessment measures, or the use of the original questionnaire, should be avoided in clinical trials because the objective propriety of evaluation is not guaranteed.

##### Compliance of cancer treatment and treatment outcomes

Information on compliance with cancer treatment and treatment outcomes is necessary to ensure that a novel intervention against OM has no detrimental effects on cancer treatment. Compliance with cancer treatment includes dose intensity in pharmacotherapy, completion rate, rate of unplanned breaks, and duration of unplanned breaks in RT. Treatment outcomes regarding survival include overall survival and cancer treatment‐related death.

##### Contents of secondary supportive care and medical resources for OM

Although a novel experimental intervention is investigated in a clinical study of OM, assessment of secondary supportive care and medical resources to treat OM may be required, such as dose and duration of opioid use, nutritional intervention, including percutaneous endoscopic gastrostomy (PEG), and total parental nutrition. From a socioeconomic perspective, the cost of the intervention and duration of hospitalization may be recorded. All the abovementioned parameters are objective and can be represented numerically.

##### The overall adverse events rather than OM

Adverse events other than OM attributable to cancer treatment should be assessed, including mucosal infection, aspiration, bacteremia, bleeding, malnutrition, and dehydration. Notably, it is recommended that any specific adverse events induced by a novel experimental intervention for OM be recorded.

##### QOL

Common symptoms related to OM considerably worsen the QOL and nutritional status of HNC patients. The evaluation of QOL plays an important role in all studies of supportive care in cancer and is generally set as one of the secondary endpoints in clinical trials of OM. The use of validated assessment measures is encouraged to evaluate global health status. Our expert panel proposed the use of a combination of the European Organization for Research and Treatment of Cancer (EORTC) Quality of Life Questionnaire–Core 30 module (QLQ‐C30) and EORTC QLQ‐OH15. Global health status and generic QOL aspects are evaluated using EORTC QLQ‐C30. By contrast, the EORTC module QLQ‐OH15 is a short and validated assessment measure that focuses on oral problems.^33^ The EORTC QLQ‐OH15 module comprises a 15‐item questionnaire that contains one 8‐item OH‐QoL scale, 3 single items (sticky saliva/mouth soreness/sensitivity to food/drink), and two 2‐item contingency scales regarding the use (yes/no), problems with dentures and reception of (yes/no), and satisfaction with information. These assessment tools have been translated into other languages, such as Japanese. To use these measures, investigators must obtain permission from the EORTC Quality of Life Group (https://qol.eortc.org/questionnaires/). No fee is required for academic use, but a fee is requested depending on the number of patients in the study if the study has commercial sponsorship.

### 3.8 Eligibility criteria

Because the ultimate goal of clinical studies in supportive care is to disseminate and implement the obtained findings to real‐world patient populations in clinical practice, the eligibility criteria should not be too restrictive. However, any factors that greatly influence the primary endpoints and assessment measures should be set as subject inclusion or exclusion criteria in OM research, as described in Section 3.5.

#### 3.8.1 Subject inclusion criteria

If the type or dose of pharmacotherapy, type of RT, irradiation dose, dose fraction, field, and radiation technique are the major risk factors of OM, these regimens for cancer treatment and their administration dose should be listed in the inclusion criteria.

#### 3.8.2 Subject exclusion criteria

General status, oral hygiene conditions, and underlying oral diseases may greatly influence the pathophysiology of the oral mucosa. If there is such a concern, the necessity of excluding these factors from the study criteria should be well discussed.

If nutritional interventions are included in the endpoints of the study, patients with gastrointestinal tract impairment and contraindications to PEG in whom invasive nutritional interventions are impossible should be excluded from the study population. If pain assessment is selected as the primary endpoint, it is recommended that patients receiving opioid treatment for the management of cancer pain episodes are excluded, or baseline use of opioids is well balanced between arms in a randomized study.

### 3.9 Research design

A study protocol on OM clinical research should be systematically organized, including endpoints, eligibility criteria, assessment measures, research design, and minimally recommended intervention. Table 3 demonstrates the examples of trial design in clinical trials associated with OM induced by RT or CRT for HNC patients.

**TABLE 3:** Examples of trial design in clinical trials associated with oral mucositis induced by radiotherapy or chemoradiotherapy for patients with head and neck cancer

Study	Sample size (N)	Cancer treatment	Assessment measures of oral mucositis	Agents	Approach	Phase of clinical trials	Primary endpoint
Zenda et al.^40^	101	CRT	CTCAE version 3.0	Opioid	Pain control	Phase 2	Compliance with RT
Le et al.^55^	188	CRT	WHO grade	Palifermin	Keratinocyte growth factor	Phase 3	Incidence of grade ≥3 OM
Gouvêa de Lima et al.^56^	75	CRT	CTCAE version 2.0	Low‐level laser therapy	Pain control/anti‐inflammatory/wound healing	Phase 3	Incidence of grade ≥3 OM, Compliance with RT
Antunes et al.^57^	94	CRT	WHO grade and OMAS scales	Low‐level laser therapy	Pain control/anti‐inflammatory/wound healing	Phase 3	Incidence of grade ≥3 OM
Leenstra et al.^58^	155	RT alone, CRT	?	Doxepin Rinse	Mouth washing agents/pain control	Phase 3	AUC for mouth and throat pain reduction
Yokota et al.^26^	120	CRT	CTCAE version 3.0	–	Oral care	Phase 2	Incidence of grade ≥3 OM (clinical exam and functional/symptomatic)
Yokota et al.^59^	97	CRT	CTCAE version 3.0	Rebamipide liquid	Mouth washing agents	Randomized phase 2	Incidence of grade ≥3 OM (clinical exam)
Yokota et al.^60^	35	CRT	CTCAE version 3.0	HMB/Arg/Gln	Nutrition support	Phase 2	Incidence of grade ≥3 OM (functional/symptomatic)
Chitapanarux et al.^61^	60	CRT	OMAS scales	Benzydamine HCl	Pain control, mouth washing agents	Randomized control trial	OMAS scales
Lozano et al.^62^	84	CRT or BRT	RTOG	Melatonin	Oral gel	Randomized phase 2	Incidence of grade ≥3 OM
Anderson et al.^63^	223	CRT	WHO scale	GC4419 (Avisopasem manganese)	Inhibitor of superoxicide	Randomized phase 2	Incidence of grade ≥3 OM

Abbreviations: AUC, area under the curve; BOC, basic oral care; BRT, the addition of cetuximab to radiotherapy; CRT, chemoradiotherapy; OM, oral mucositis; RT, radiotherapy.

Clinical practice guidelines for the management of mucositis secondary to cancer therapy issued by the Multinational Association of Supportive Care in Cancer and International Society of Oral Oncology (MASCC/ISOO)^34^ recommend the use of cryotherapy, photobiomodulation therapy, growth factors, and cytokines for specific OM. Furthermore, the presence or absence of intervention by dentists often greatly influences the outcomes of OM in basic oral care. Because these interventions have a big impact on the evaluation of the OM severity, it is essential to eliminate deviations in the extent of these interventions between the control and experimental arms in randomized trials. Unless these interventions are mandatory in randomized trials, their presence or absence should be set as stratification factors.

### 3.10 Supplement: minimally recommended intervention in OM research

Published recommendations for mucositis interventions include MASCC/ISOO,^34^ National Comprehensive Cancer Network (NCCN),^35^ and a Cochrane review. Multidisciplinary strategies based on systematic oral care, pain control, and additional nutritional interventions are required to reduce the OM burden.

#### 3.10.1 Basic oral care

Maintenance of oral hygiene is designated as basic oral care (BOC) in this research policy. Although the recommended grade of BOC is not high in the clinical practice guidelines by the Mucositis Study Group of MASCC/ISOO,^34^ there is adequate positive consensus to support a suggestion in favor of performing BOC for the prevention of OM across all cancer treatment modalities. The NCCN guidelines and a National Cancer Institute report also recommend BOC as a standard practice to prevent infections and alleviate mucosal symptoms.^35^, ^36^

Regardless of the novel treatment chosen for OM, BOC provided by a multidisciplinary oral care team, composed of physicians, dentists, dental hygienists, nurses, pharmacists, and dieticians, is essential. The outcome of OM is highly influenced by BOC providers. In particular, interventions by dentists play an important role in the outcome. Before cancer treatment, patients should undergo routine oral screening by dentists to determine the status of the teeth, periodontal tissue, and oral hygiene. Professional dental treatment may be performed if necessary. Patients should be informed about the adverse oral reactions caused by cancer treatment and provided instructions about tooth brushing, mouth washing, nutrition, and lifestyle. BOC and OM assessments should be performed and documented continuously by medical staff. Furthermore, oral care programs may enhance patients' self‐care abilities to perform BOC and symptom control.^37^, ^38^, ^39^

#### 3.10.2 Pain control

Substantial pain induced by OM interferes with the patients' ability to chew and swallow, impairs the patients' QOL, and results in the loss of patients' motivation for cancer therapy. Therefore, systematic management of OM‐induced pain is indispensable.

A pain control program has been developed as supportive care for OM. Oral administration of mucoprotective agents, acetaminophen, fast‐acting, and long‐acting opioids, and mouthwash liquid containing local analgesic agents are convenient for the management of pain induced by pharmacotherapy for solid tumors and RT or CRT for HNC in outpatient wards.^40^ Administration access for patients unable to swallow or who undergo enteral nutrition (EN) includes a feeding tube, such as a nasogastric tube or PEG. Continuous infusion of opioids and a fentanyl patch are also available as alternative access. In stem cell transplantation, all patients are treated with continuous intravenous infusions on an inpatient basis. Therefore, intravenous administration of opioids is more common than oral administration of opioids.

#### 3.10.3 Nutrition support

There is an algorithm for the use of parenteral nutrition and EN in the American Society for Parenteral and Enteral Nutrition (ASPEN) clinical guidelines.^41^, ^42^ The route of nutritional support is largely determined by the anticipated duration of nutritional support, prognosis, and the ability to pass through the upper digestive tract.

##### Oral intake

Oral intake is highly encouraged, even in the presence of OM. The food style, including fluid, liquid, or solid, maybe individually arranged according to the OM severity. Nutritional support supervised by a dietitian is recommended.

##### Enteral nutrition

Approximately half of the HNC patients (34%–57%) receiving CRT experience grade ≥3 mucositis,^43^ which is often accompanied by inadequate oral intake. Thus, prophylactic PEG has been widely performed before CRT for HNC.^44^, ^45^, ^46^, ^47^ However, PEG placement should be avoided during CRT because of the increased risk of PEG‐related complications, such as infection and subsequent RT interruption. Insertion of a nasogastric tube is also available during CRT.

##### Parenteral nutrition

Parenteral nutrition (PN) is a common nutritional approach for patients with hematological malignancies. However, care must be taken when using parenteral nutrition in CRT for HNC and esophageal cancer if a central venous access device is placed within the irradiated region.

## 4 DISCUSSION

MASCC/ISOO has released the clinical practice guidelines for the management of mucositis secondary to cancer therapy.^15^, ^34^ They applied the Hadorn criteria,^48^ which were modified to meet the requirements of mucositis studies,^49^ to evaluate scientific evidence. The recently updated version (2019–2020) also uses it.^50^ Therefore, MASCC/ISOO potentially recommends satisfying the modified Hardon's criteria. However, no recommendations on appropriate methodological approaches for clinical research have been reported in the field of OM research. Specifically, the merit and demerit in each OM assessment measure are not systematically organized. Therefore, the reality is that OM assessment measures have been quite variable among studies, which makes it quite challenging to compare the results among studies. Based on the general research policy we have published,^3^ our expert panel developed the first specific clinical research policy on OM, rather than the methodology of OM management in the clinical practice. This policy covers the conduct of research on not only minimally invasive nursing interventions, such as basic oral care, but also medical interventions, including systemic pharmacotherapies, local mouth‐wash agents, and nutritional support.

The contents of the policy were systematically organized according to the items in the study protocol, including endpoints, eligibility criteria, assessment measures, and research design. Since the assessment of OM is subjective and sometimes lacks reproducibility, this policy primarily focuses on recommendations on how to set endpoints and assessment measures in OM research from the aspect of CRO and PRO. Notably, we highlighted the assessment of functional disorders and oral symptoms related to OM, including swallowing dysfunction, disability of oral intake, and pain, which may realistically reflect patients' QOL. Furthermore, OM is acknowledged as the principal limiting factor for further intensification of cancer treatment.^51^ Treatment interruptions and dose reductions due to OM may ultimately affect therapeutic outcomes, including cure rates, the durability of cancer remission, and patient survival.^52^, ^53^, ^54^ Therefore, compliance with cancer treatment and treatment outcomes should be designated as important endpoints in OM research.

A limitation of this study is that the policy was exclusively developed by Japanese supportive care specialists. However, this policy has been certified through critical review by several scientific societies. The board members of this policy had sufficient experience as principal investigators in several prospective trials. Furthermore, all the assessment tools we mentioned are recognized as global standards in the field of OM research.

In conclusion, we have established a novel research policy for OM. We hope that this policy will be referred to in the field of OM research to conduct high‐quality clinical studies and report reliable evidence.

## AUTHOR CONTRIBUTIONS

All authors have made substantial contributions to conception and design and have been involved in drafting the manuscript and revising.

## CONFLICT OF INTEREST

There is no conflict of interest for this manuscript among our expert panel.

## ETHICS STATEMENT

Not applicable to this article as this is a research policy.

## Supporting information


**Appendix S1** Supplementary Information

## Data Availability

Data sharing is not applicable to this article as no new data were created or analyzed in this study.
