# Supplementary material for: J‐SUPPORT research policy for oral mucositis associated with cancer treatment
Source: Cancer Med. 2022 Jun 12;11(24):4816–29. doi: 10.1002/cam4.4811 (PMC9761062; doi:10.1002/cam4.4811)
Supplement: Supplementary file 1 — Appendix S1 Supplementary Information [file CAM4-11-4816-s001.docx]

## Bottom line

## Research policy for oral mucositis associated with cancer treatment

## Guideline Question

How should clinical research on oral mucositis (OM) associated with cancer treatment be designed and conducted?

## Target Audience

Any researchers who are planning and conducting clinical trials for the management of oral mucositis associated with cancer treatment.

## Methods

An expert panel was convened to develop clinical research policy in the area of oral mucositis associated with cancer treatment on the basis of review of the medical literature and clinical experience.

## Key Recommendations

#### Definition of OM in this research policy

*Qualifying statement.*

- This policy targets the research area of OM, defined as injury to the oral mucosa associated with cancer treatment, including cancer pharmacotherapy and radiotherapy, but not the research area of chronic graft-versus-host disease (GVHD)-related OM, and OM in terminally ill cancer patients.

#### Type of OM

##### Recommendation 2.1. OM associated with radiotherapy (RT)

- Researchers should confirm the radiation dose, field, and the concurrent use of cytotoxic and targeting agents in planning clinical studies of OM associated with RT.

##### Recommendation 2.2. OM associated with pharmacotherapy

- Researchers should understand that cytotoxic chemotherapeutic agents and molecular targeting agents, including, inhibitors of mammalian target of rapamycin, tyrosine kinase inhibitors, and immune checkpoint inhibitors, induce OM.
- OM can occur as a severe drug-related symptom of Stevens-Johnson syndrome.

#### Characteristics of OM

*Recommendation 3.* *Common mucosal findings*

- Researchers should be aware of common mucosal findings in OM.
- Researchers should understand local and systemic impact of OM. The occurrence of OM has significant impacts on completion of cancer treatment.

#### Characteristics of OM research

##### Recommendation 4.1.

- A multidisciplinary approach involving physicians, nurses, pharmacists, dentists, dental hygienists, dieticians, speech therapists, and physical therapists is required in the management of OM.

##### Recommendation 4.2.

- Researchers should be aware of the potential benefits and risks of intervention against OM. A novel experimental intervention / supportive care should not have detrimental effect on cancer treatment.

##### Recommendation 4.3.

- The duration of a study on OM should be determined in accordance with the type of cancer treatment and the anticipated duration of OM.

#### Risk factors for OM

##### Recommendation 5.

- Researchers should assess both host- and cancer treatment-related risk factors for OM in the target population for OM research.
- The risk factors for OM may determine subject inclusion or exclusion criteria in a study (see Section 8).

#### Target population for OM research

##### Recommendation 6.

- The content of cancer treatments should be as homogeneous as possible within the study cohort.
- Researchers should set the factors regarding cancer treatments as stratification factors, if different treatment regimens are used in randomized trials.

##### Recommendation 6.1. Patients undergoing RT in head and neck region

- Patients undergoing chemoradiotherapy and RT alone in head and neck region should not be the same population target.
- The radiation technique should be the same in a target population.
- Concurrent pharmacotherapy in combination with RT should be the same within the study cohort

##### Recommendation 6.2. Patients treated by pharmacotherapy

- The dose and dosing schedule of pharmacotherapy should be the same within a target population.

##### Recommendation 6.3. Recipients of hematopoietic stem cell transplantation

- The content of chemotherapy and total body irradiation should be the same in the target population in hematopoietic stem cell transplantation recipients.

#### Endpoints and assessment measures in OM research

##### Recommendation 7.1. General remarks

- Researchers should assess both clinician-reported outcomes (CRO) and patient-reported outcomes (PRO) in OM research. In randomized trials, researchers should use common assessment measures between the study arms, in terms of observers of OM and the procedure of OM measurement.
- Researchers should stipulate the observation period and frequency in the protocol.

##### Recommendation 7.2. Assessment measures in the clinical study of OM

- Primary endpoints should be set depending on the timing and aspects that investigators aim to improve the outcome of OM in the study.

###### Recommendation 7.2.1.

- Researchers should understand assessment of OM severity by medical staff (clinicians) and by patients.

Recommendations 7.2.1.1. Clinician-Reported Outcomes: CROs

- CRO assessment measures are classified into those assessing functional disorders and symptoms induced by OM, and those assessing mucosal findings.
- The degree of importance placed on the functional/symptomatic aspects of OM or mucosal findings differ among the scales. A list of assessment scales for severity of oral mucositis in the clinical study can be found in Table 1 and 2.
- Researcher should choose CRO assessment measures, depending on the primary purpose of the clinical trials.
- Medical staff working on OM observation should undergo specific training and be qualified. The number of OM observers was limited by careful selection.
- Researchers should organize independent central review committee (ICRC) for objective assessment of mucosal findings. ICRC members should be independent of the observers of the oral mucosa and be blinded to patient clinical outcome data.
- Observation of the oral mucosa and subsequent determination of OM grading should be performed separately.
- In the process of central review, photographic data of the oral mucosa uploaded by investigators or the Oral Mucositis Assessment Sheet [27] may be available. To maintain consistent photographic quality, the procedure of taking photographs should be standardized in the protocol.
- The study protocol should define the endpoints assessed by CRO assessment measures, the data analysis plan, and their interpretation. Systematic oral observation procedures should be clarified in the protocol or standard operating procedures.

Recommendations 7.2.1.2. Patient-Reported outcomes: PROs

- The PRO measurement items in clinical trials of OM include pain, swallowing difficulty, dry mouth, and taste changes.
- Our expert panel recommended collecting patient self-reports using PRO-CTCAE.
- All items were individually selected from the PRO-CTCAE according to the purpose of each clinical trial. Refer to the Consolidated Standards of Reporting Trials (CONSORT) PRO and Standard Protocol Items: Recommendations for Interventional Trials (SPIRIT)-PRO.
- The visual analog scale (VAS), numeric rating scale (NRS), face scale, and support team assessment schedule (STAS) are used to assess pain induced by OM.
- The method of PRO measurements should be the same between the study arms, and the data analysis plan should be clearly specified in the protocol when designing the trial.
- PRO assessment measures should be used in accordance with the manual. Capturing and editing of validated PRO assessment measures, or the use of the original questionnaire, should be avoided in clinical trials.

###### Recommendation 7.2.2. Compliance of cancer treatment and treatment outcomes

- Researchers should evaluate compliance with cancer treatment and treatment outcomes to ensure that a novel intervention against OM has no detrimental effects on cancer treatment.

###### Recommendation 7.2.3. Contents of secondary supportive care and medical resources for OM

- Researchers should assess secondary supportive care and medical resources to treat OM (e.g., dose and duration of opioid use, nutritional intervention).
- From a socioeconomic perspective, the cost of the intervention and duration of hospitalization may be recorded.

###### Recommendation 7.2.4. The overall adverse-events rather than OM

- Researchers should assess adverse events other than OM attributable to cancer treatment and a novel experimental intervention for OM.

###### Recommendation 7.2.5. quality of life (QOL)

- QOL assessment by using validated questionnaires in conducting OM-research is highly encouraged.
- Our expert panel proposed the use of a combination of the European Organization for Research and Treatment of Cancer (EORTC) Quality of Life Questionnaire–Core 30 module (QLQ-C30) and EORTC QLQ-OH15.

#### Eligibility criteria

##### Recommendation 8.

- The eligibility criteria of OM-research should not be too restrictive. However, researchers should set any factors that greatly influence the primary endpoints and assessment measures as subject inclusion or exclusion criteria.

##### Recommendation 8.1. Subject inclusion criteria

- Researcher should list the following factors in the subject inclusion criteria; the type or dose of pharmacotherapy, type of RT, irradiation dose, dose fraction, field, and radiation technique, if these are the major risk factors of OM.

##### Recommendation 8.2. Subject exclusion criteria

- Researcher should discuss the necessity of excluding the subjects with poor general status, poor oral hygiene conditions, and underlying oral diseases.
- Researcher should exclude the subjects with gastrointestinal tract impairment and contraindications to PEG in whom invasive nutritional interventions are impossible, if nutritional interventions are included in the endpoints of the study.
- Researcher should exclude the subjects receiving opioid treatment for the management of cancer pain episodes, if pain assessment is selected as the primary endpoint. Alternatively, baseline use of opioids should be well balanced between arms in a randomized study.

#### Research design

##### Recommendation 9.1.

- A study protocol on OM clinical research should be systematically organized, including endpoints, eligibility criteria, assessment measures, research design, and minimally recommended intervention.

##### Recommendation 9.2.

- Clinical practice guidelines recommend several managements of OM and intervention by dentists. Researchers should eliminate deviations in the extent of these interventions between the control and experimental arms in randomized trials. Alternatively, researchers should set the presence or absence of these interventions as stratification factors.

#### Supplement: minimally recommended intervention in OM research

##### Recommendation 10.

- Multidisciplinary strategies based on systematic oral care, pain control, and additional nutritional interventions are recommended to reduce the OM burden regardless of the type of novel experimental intervention.

##### Recommendation 10.1. Basic Oral Care

- Maintenance of oral hygiene by basic oral care is recommended to prevent infections and alleviate mucosal symptoms. Basic oral care provided by a multidisciplinary oral care team, including dentists, is highly recommended.

##### Recommendation 10.2. Pain control

- Systematic management of OM-induced pain is indispensable. Clinicians may prescribe mucoprotective agents, acetaminophen, fast-acting and long-acting opioids, and mouthwash liquid containing local analgesic agents for the management of OM-induced pain.

##### Recommendation 10.3. Nutrition Support

- Clinicians should understand algorithm for the use of parenteral nutrition and enteral nutrition. The route of nutritional support includes oral intake, enteral nutrition, and parenteral nutrition, and is largely determined by the modality of cancer treatment, the anticipated duration of nutritional support, prognosis, and the ability to pass through the upper digestive tract.
